# The role of exercise training on lipoprotein profiles in adolescent males

**DOI:** 10.1186/1476-511X-13-95

**Published:** 2014-06-09

**Authors:** Majid S Koozehchian, Farzad Nazem, Richard B Kreider, William J Roberts, Thomas M Best, Yi Rong, Li Zuo

**Affiliations:** 1 Exercise and Sport Nutrition Laboratory, Department of Health & Kinesiology, Texas A&M University, College Station, TX 77843, USA; 2 Molecular Physiology and Rehabilitation Research Laboratory, Radiologic Sciences and Respiratory Therapy Division, School of Health and Rehabilitation Sciences, The Ohio State University College of Medicine, The Ohio State University Wexner Medical Center, Columbus, OH 43210, USA; 3 Division of Sports Medicine, Department of Family Medicine, Sports Health & Performance Institute, The Ohio State University, Columbus, OH 43210, USA; 4 Department of Radiation Oncology, The Ohio State University College of Medicine, The Ohio State University Wexner Medical Center, Columbus, OH 43210, USA; 5 Department of Health & Kinesiology, Bu Ali Sina University, Hamedan 65174, Iran

**Keywords:** Cardiovascular risk, Anti-risk factors, Cholesterol, Lipids

## Abstract

**Background:**

Major cardiovascular disorders are being recognized earlier in life. In this study we examined the effects of swimming and soccer training on male adolescent lipid-lipoprotein profiles relative to a maturity matched control group to determine the effects of these exercises on specific cardiovascular risk and anti-risk factors.

**Methods:**

Forty five adolescent males (11.81 ± 1.38 yr) including swimmers (SW), soccer players (SO), and non-athlete, physically active individuals as controls (C), participated in this study. Training groups completed 12-wk exercise programs on three non-consecutive days per week. Plasma low-density lipoprotein (LDL), very low density lipoprotein (VLDL), high density lipoprotein (HDL), apolipoprotein A-I (apoA-I), apolipoprotein B (apoB), total cholesterol (TC), and triglyceride (TG) levels were measured in control, pre-training, during-training, and post-training.

**Results:**

In response to the 12-wk training period, the SO group demonstrated a decrease in the mean LDL level compared to the SW and C (SW: 0.15%; SO: −9.51%; C: 19.59%; *p <* 0.001) groups. There was an increase in both the SW and SO groups *vs*. the control in mean HDL (SW: 5.66%; SO: 3.07%; C: −7.21%; *p <* 0.05) and apoA-I (SW: 3.86%; SO: 5.48%; C: −1.01%; *p* < 0.05). ApoB was considerably lower in the training groups *vs.* control (SW: −9.52%; SO: −13.87%; C: 21.09%; *p* < 0.05). ApoA-I/apoB ratio was significantly higher in training groups vs. control (SW: 16.74%; SO: 23.71%; C: −17.35%; *p* < 0.001). There were no significant differences between groups for other factors.

**Conclusions:**

The favorable alterations in LDL, HDL, apoA-I, and apoB observed in the training groups suggest that both regular swimming or soccer exercise can potentially mitigate cardiovascular risk in adolescent males.

## Background

In 2006, the World Health Organization reported that the death rate attributed to cardiovascular diseases (CVDs), the number one cause of death globally, was 262.5 per 100,000. CVDs are linked to numerous risk factors, including tobacco use, unhealthy diet, obesity, physical inactivity, hypertension, diabetes, and abnormal lipid profiles [1]. Though major cardiovascular events typically occur in adults, it has been demonstrated that the associated risk factors often develop early in life. For instance, atherosclerosis is the leading cause of coronary heart disease (CHD; one type of CVD) which can begin early in childhood and advance into adulthood [2,3]. In addition, children of parents diagnosed with CVD are known to exhibit a higher probability of developing CVD at a much earlier age compared to others [3,4]. Fatty streaks in the aorta may also be present as early as three years of age and fibrous plaques have been observed during adolescence, both of which are associated with atherosclerosis [2]. Since previous studies have illustrated that a considerable number of school-age children have risk factors that are predictive of CHD in adults [5,6], the early years of life may be a critical time period to reduce the risk of CHD [1,7,8].

Several longitudinal studies, beginning from childhood, show that serum lipids and lipoproteins are important indicators of CHD development in adulthood [9,10]. For example, the presence of CHD in adults increases with elevated levels of low-density lipoprotein-cholesterol (LDL-C) and decreases with elevated levels of high-density lipoprotein-cholesterol (HDL-C) [9]. The role of LDL-C as a major predictor of CHD is well established, and an increase in its levels has been shown to cause progression of atherosclerotic plaque formation [11]. Furthermore, previous findings demonstrate a clear relationship between CHD and total cholesterol (TC) levels [12]. Thus, the TC/HDL-C ratio has been proposed as another indicator of risk for CHD [12,13]. Moreover, an overall reduction in HDL-C and apolipoprotein A-I (apoA-I) in CHD subjects compared with controls is highly correlated with triglyceride (TG) levels and CHD risk [14,15]. Apolipoproteins, which are another important marker for CHD risk, may enhance CHD evaluation over conventional lipids [16]. Specifically, the ratio of apolipoprotein B (apoB) and apoA-I has been considered a strong CVD risk marker/factor due to the interaction of these proteins with LDL and HDL respectively [16]. As expected, higher levels of cardiovascular anti-risk factors and lower levels of risk factors have been consistently reported for more active or fit individuals [17].

The chronic cardiovascular adaptations to regular exercise training in adults are well documented. However, whether adolescents can improve health markers after training before puberty is still unclear [18]. There is also a lack of similar reports on adolescents and exercise training to change lipid profiles. Exercise is one of the most effective approaches to maintain optimal blood lipid levels in adolescents [19]. Related studies have indicated that training decreases LDL and TG levels as well as the TC/HDL ratio in children [13,20]. Yet the type, intensity, and volume of exercise responsible for these results is still unknown [8]. Therefore, the purpose of this study was to compare the effect of two common exercise training programs on well-established cardiovascular risk and anti-risk factors in adolescent males.

## Methods

### Study participants

The study protocol was approved by the Bu Ali Sina University Medical Ethics committee. Written informed consent was obtained from the parents (both parents if possible) and the adolescents themselves. Forty five adolescent males (age: 11.8 ± 1.38 yr; height: 149 ± 8.38 cm; weight: 40 ± 7.8 kg) volunteered to participate in the study. Swimmers (SW, n = 15) were recruited from the Shiroodi sports complex, soccer players (SO, n = 15) were recruited from the Heydarnia sports complex, and controls (C, n = 15) were recruited from four schools located in Tehran, Iran. The training groups were involved in the related sport activity (swimming or soccer) for at least three months immediately prior to the initiation of the study. Controls were physically active, but they were not involved in any specific type of training. All participants were recognized to be asymptomatic and were not using any type of medication known to influence lipid and lipoprotein profiles. Prior to the training program, the lipid levels were between normal concentrations in all three groups following the parameters set by Szamosi *et al.*[21].

### Training program

The 12-week exercise training programs, including swimming (SW) and soccer (SO), were conducted in the Shiroodi pool and the Heydarnia sports complex, respectively. The rationale of such a training profile was based on previous recommendations that the exercise should 1) involve large muscle groups, 2) be continuous in nature, and 3) be conducted for at least 12 weeks [22]. Each week, the SW group participated in a three day/week training program. All three days involved a 60-min supervised swim. Each session began with 15–20 min of free style, in which the boys swam in freestyle for short distances (up to 200 m) separated by short breaks. The remainder of the session (40–45 min) was devoted to breaststroke and backstroke. Although the swim distance was longer for these strokes (400 m), the activity included a few short breaks to try and match soccer in terms of intensity and rest to exercise cycle. SO participants took part in a 3 day/week supervised soccer training program, with one 60-minute session on each of the three days. A training week consisted of warm up, stretch, endurance running, small team games, soccer techniques, strength training, and match play.

### Blood samples and analysis

Written instructions were given to the parents illustrating the importance of fasting for 12 hr before blood sampling, and this was verbally reemphasized to the participants before the testing time. Participants were asked not to be involved in any high intensity physical activity during the 48 hr before testing. A 10 mL blood sample was drawn from the antecubital vein using a butterfly needle from overnight fasted participants after 48 hr since the last training session. The samples were collected in pre-chilled vacutainers and gently rotated several times to ensure proper mixing. Plasma samples were obtained after centrifugation at 3,000 RPM and 4°C for 15 min. Samples were coded and immediately transported to the laboratory for analysis of lipid and lipoprotein profiles.

All lipid and lipoprotein determinations were performed in duplicate in the same laboratory. ApoA-I and apoB were measured by immunoturbidimetry [23]. Standard enzymatic methods were used to determine serum TC (Boenhringer CHOD-PAP kits, Boenhringer Mannheim GmbH, Mannheim, Germany) and TG (Boenhringer Mannheim GmbH). HDL was measured by dextran sulfate-magnesium precipitation [24], followed by enzymatic determination of TC. The plasma LDL concentration was calculated using the Friedwald formula [25]. The very low density lipid (VLDL) cholesterol level was estimated from TG levels [26].

### Statistical analysis

We applied the test for normality of the variables. The statistical power of this study was 0.8. Thus, in this study, three groups were involved and were tested four times. Accordingly, the optimal test was repeated measures as MANOVA. Results are expressed as mean ± SD. A mixed model repeated measures MANOVA was used to identify significant differences in mean LDL, HDL, VLDL, apoA-I, apoB, TC, TG, and the ratios of TC/HDL and apoA-I/apoB values across all groups. Multiple comparisons by LSD *post hoc* test were used to highlight the significant differences. The significant difference level was set at *p* < 0.05. All statistical analyses were performed using SPSS for Windows (Release 22 Chicago, IL, USA). With an acceptance of a dropout of two participants in the SO group and one in the SW group, the sample size of the study provided 90% statistical power to detect differences in the blood lipid profile of groups at a significance level of *p* < 0.05.

## Results

Fourteen of the swimmers and thirteen of the soccer players completed their training program and were included in the analyses. Three participants dropped out of the exercise groups because they missed several training sessions. There was no drop in the control group (C).

The physical characteristics of the SW, SO, and C groups before and after the 12-week period are presented in Table 1. The three groups were matched for anthropometric parameters, age, and pubertal stages (Table 1). After the 12-week training programs, resting heart rate (RHR) markedly decreased in SO vs. SW and control groups (*p* < 0.05; Table 1). No significant changes were found for body mass, BMI, body fat mass, waist circumference, hip circumference, waist/hip ratio, systolic blood pressure, and diastolic blood pressure between the groups (Table 1).

**Table 1:** Mean values of male adolescent physical characteristics across the three groups pre- and postintervention

**Variable**	**G**	**0 weeks**	**4 weeks**	**8 weeks**	**12 weeks**	**P**
Body mass, kg	**SW**	42.58 ± 7.67	43.09 ± 8.28	43.69 ± 7.84	44.17 ± 8.33	*p* > 0.05
	**SO**	37.17 ± 7.58	37.86 ± 7.35	38.07 ± 7.35	38.52 ± 7.60	
	**C**	36.18 ± 6.09	36.60 ± 6.01	37.05 ± 6.32	37.66 ± 6.23	
BMI (kg/m^2^)	**SW**	18.60 ± 2.30	19.52 ± 2.58	18.65 ± 2.18	17.54 ± 2.41	*p* > 0.05
	**SO**	17.71 ± 2.16	18.76 ± 4.65	16.64 ± 2.50	17.60 ± 4.35	
	**C**	17.75 ± 2.32	17.67 ± 1.32	16.62 ± 2.38	18.66 ± 3.23	
Body fat mass (%)	**SW**	14.94 ± 2.90	14.53 ± 4.16	14.10 ± 4.51	13.22 ± 4.83	*p* > 0.05
	**SO**	13.77 ± 4.83	14.72 ± 4.27	12.28 ± 4.59	11.75 ± 6.15	
	**C**	12.69 ± 3.24	13.57 ± 2.51	13.82 ± 3.24	15.41 ± 3.47	
Waist circumference, cm	**SW**	69.60 ± 4.87	70.57 ± 5.13	71.78 ± 5.27	65.10 ± 5.60	*p* > 0.05
	**SO**	67.30 ± 6.15	69.11 ± 5.56	70.15 ± 5.90	71.26 ± 6.62	
	**C**	65.26 ± 5.95	65.36 ± 5.68	66.76 ± 5.51	67.60 ± 5.86	
Hip circumference, cm	**SW**	73.35 ± 4.99	73.39 ± 4.92	74.64 ± 5.58	75.57 5.35	*p* > 0.05
	**SO**	70.19 ± 6.95	70.07 ± 5.34	70.80 ± 6.30	71.61 ± 6.65	
	**C**	68.40 ± 5.76	68.93 ± 5.74	70.50 ± 5.89	70.5 ± 5.82	
WH ratio	**SW**	0.94 ± 0.01	0.95 ± 0.04	0.95 ± 0.04	0.94 ± 0.06	*p* > 0.05
	**SO**	0.95 ± 0.02	0.98 ± 0.01	0.98 ± 0.05	0.99 ± 0.06	
	**C**	0.95 ± 0.04	0.94 ± 0.05	0.94 ± 0.05	0.95 ± 0.06	
SBP, mmHg	**SW**	117.29 ± 3.29	116.00 ± 2.82	114.57 ± 2.87	115.00 ± 3.11	*p* > 0.05
	**SO**	120.85 ± 3.31	116.46 ± 4.55	118.08 ± 3.47	118.31 ± 3.90	
	**C**	117.60 ± 2.64	116.13 ± 4.10	115.60 ± 3.39	113.60 ± 3.13	
DBP, mmHg	**SW**	64.00 ± 5.14	62.86 ± 3.11	63.71 ± 2.81	63.14 ± 2.79	*p* > 0.05
	**SO**	64.00 ± 3.16	64.15 ± 3.87	64.46 ± 3.07	63.08 ± 3.70	
	**C**	64.13 ± 3.06	63.87 ± 3.81	62.80 ± 2.59	61.20 ± 2.80	
RHR, beats/min	**SW**	74.71 ± 9.23	69.43 ± 7.90	66.43 ± 8.23	67.29 ± 7.30	*p* < 0.05
		**SO**	69.60 ± 17.90	66.93 ± 10.08	65.87 ± 7.72	64.60 ± 7.82*
		**C**	76.15 ± 9.78	74.15 ± 9.78	71.85 ± 8.62	72.69 ± 7.99

Data are means ± SD. *p < 0.05 represents a significant difference between groups. BMI = body mass index; WH ratio = waist/hip ratio; SBP = systolic blood pressure; DBP = diastolic blood pressure; RHR = resting heart rate; G = group.

**Figure 1:**
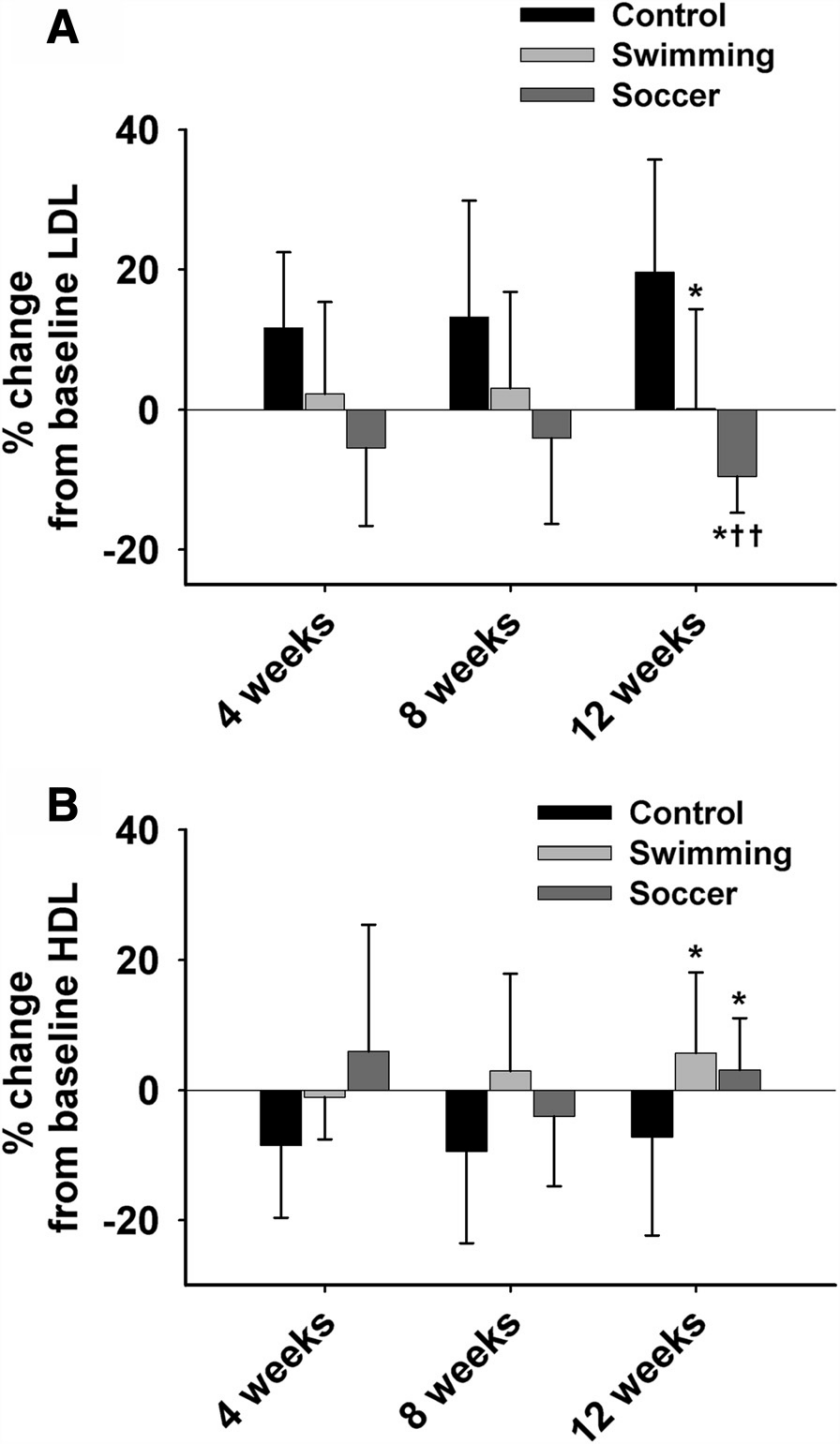
**Variation in LDL and HDL levels among the three experimental groups.** Averaged data (±SD) showing % change in LDL **(A)** and HDL **(B)** levels from baseline (0 weeks) in the control, swimming, and soccer training groups. *Significant difference between the training groups and control (*p* < 0.05). ††Significant difference from baseline within a group (*p* < 0.01).

Figure 1 indicates the % change in HDL and LDL across all three training groups throughout the 12-week period. Participation in the regular exercise training caused a decrease in the mean LDL levels in the SO group compared to the other groups (in %; SW: 0.15% ± 14.20; SO: −9.51% ± 5.21; C: 19.59% ± 16.15; *p* < 0.001). However, the LDL levels remained unchanged following the 12-week training period within the SW group and increased within the control group. An increase was observed in the mean HDL levels in both training groups vs. the control (in %; SW: 5.66% ± 12.43; SO: 3.07% ± 7.95; C: −7.21% ± 15.13; *p <* 0.05).

**Figure 2:**
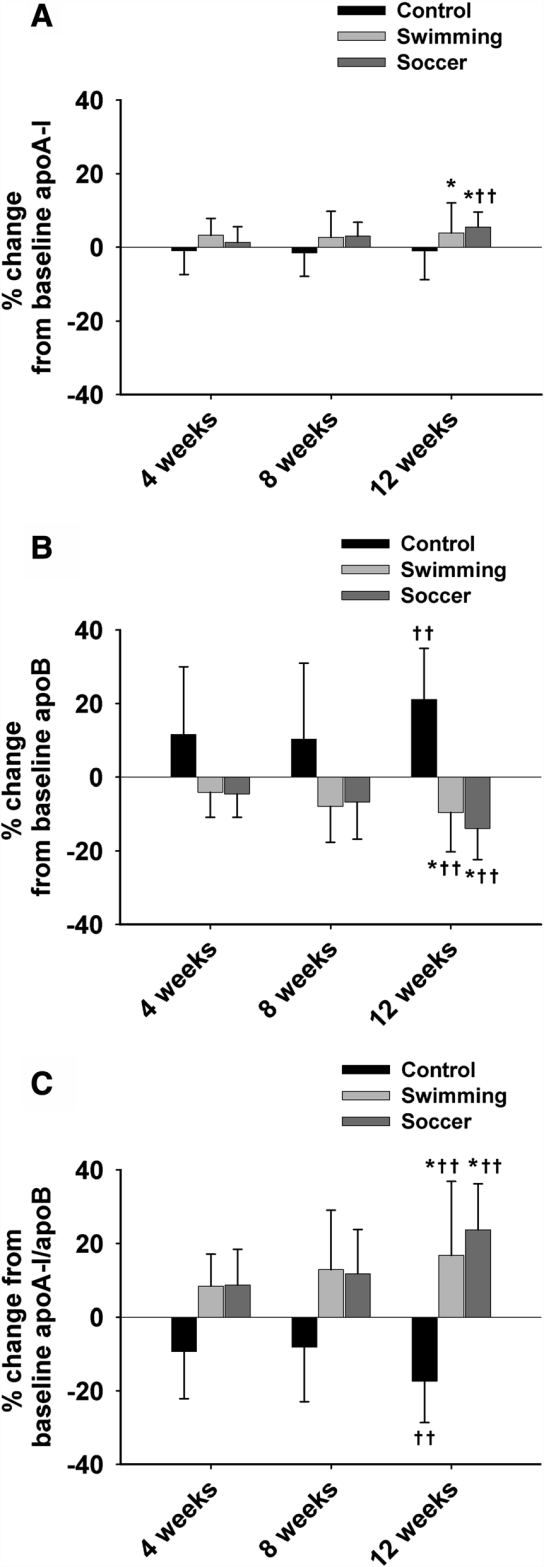
**Percent change in apoA-I, apoB, and apoA-I/apoB ratio among the three experimental groups.** Summarized mean data (±SD) demonstrating % change in apoA-I **(A)** and apoB **(B)** levels from baseline (0 weeks) in the control, swimming, and soccer training groups. **(C)** The % change from baseline of the apoA-I/apoB ratio. *Significant difference between the training groups and control (*p* < 0.05). ††Significant difference from baseline within a group (*p* < 0.01).

As shown in Figure 2, participants in both training groups experienced an increase in the mean % change from baseline apoA-I levels (in %; SW: 3.86% ± 8.18; SO: 5.48% ± 4.08; C: −1.01% ± 7.74; *p* < 0.05) as well as a considerable decrease in apoB levels compared to the control group (in %; SW: −9.52% ± 10.72; SO: −13.87% ± 8.49; C: 21.09% ± 13.88; *p* < 0.05). A significant increase in apoA-I levels from baseline was observed within the SO group (*p* < 0.05, respectively). There was a large decrease in mean % change from baseline apoB levels within the SW (*p* < 0.01) and SO groups (*p* < 0.01), while apoB levels increased for the controls (*p* < 0.001). ApoA-I/apoB ratio was significantly higher in the training groups compared to the controls after 12 weeks (in %; SW: 16.74% ± 20.12; SO: 23.71% ± 12.49: C: −17.35% ± 11.29; *p* < 0.001). Moreover, the apoA-I/apoB ratio was increased within the SW and SO groups after 12 weeks (*p* < 0.01). The apoA-I/apoB ratio was attenuated within the controls (*p* < 0.01).

As illustrated in Table 2, the training protocols had no effect on the mean TC levels (in %; SW: 1.95% ± 15.02; SO: 3.72% ± 6.07; C: −1.07% ± 11.83), TG levels (in %; SW: −1.51% ± 20.45; SO: −5.10% ± 24.97; C: 3.62% ± 9.59), VLDL levels (in %; SW: −1.51% ± 20.45; SO: −5.10% ± 24.97; C: 3.62% ± 9.59), and the mean TC/HDL ratio (in %; SW: −2.29% ± 18.68; SO: −5.87% ± 11.38; C: 6.46% ± 29.22) across the training groups (non-significant; n.s.). Note, the absolute values are shown in the table.

**Table 2:** Mean values of cardiovascular disease risk and anti-risk factors among adolescents

**Variable**	**G**	**0 weeks**	**4 weeks**	**8 weeks**	**12 weeks**	**P**
TC (mg/dl)	**SW**	161.50 ± 22.31	155.00 ± 21.40	157.86 ± 23.66	162.93 ± 21.29	*p* > 0.05
	**SO**	156.46 ± 22.50	156.54 ± 21.03	153.92 ± 20.74	149.92 ± 17.98	
	**C**	156.13 ± 24.02	153.40 ± 24.13	152.47 ± 23.19	153.47 ± 23.43	
TG (mg/dl)	**SW**	85.14 ± 23.86	82.07 ± 23.03	81.71 ± 22.10	82.21 ± 20.99	*p* > 0.05
	**SO**	73.85 ± 25.15	71.69 ± 16.42	68.69 ± 17.25	66.38 ± 19.15	
	**C**	66.93 ± 15.07	66.13 ± 14.05	66.47 ± 14.16	69.00 ± 15.08	
VLDL (mg/dl)	**SW**	17.02 ± 4.77	16.41 ± 4.60	16.34 ± 4.42	16.44 ± 4.19	*p* > 0.05
	**SO**	14.76 ± 5.03	14.33 ± 3.28	13.73 ± 3.45	13.27 ± 3.83	
	**C**	13.38 ± 3.01	13.22 ± 2.81	13.29 ± 2.83	13.80 ± 3.01	
TC/HDL ratio	**SW**	2.95 ± 0.32	2.87 ± 0.40	2.85 ± 0.48	2.87 ± 0.60	*p* > 0.05
		**SO**	2.64 ± 0.65	2.77 ± 0.79	2.75 ± 0.87	2.51 ± 0.80
		**C**	2.83 ± 0.64	3.07 ± 0.81	3.16 ± 0.93	3.01 ± 0.98

Data are means ± SD. VLDL = very low density lipoprotein; TC = total cholesterol; TG = triglyceride; HDL = high-density lipoprotein.

## Discussion

The primary findings of the current study indicate that 12 weeks of exercise increases HDL, apoA-I, and apoA-I/apoB ratio levels with two forms of training, swimming (SW) and soccer (SO). In addition, LDL levels decreased in the SO group. These lipid profile alterations may reflect a possible reduction in the prevalence of CVD in populations undergoing a training program; longitudinal studies are needed to test this attractive possibility. The significant increase in apoA-I levels in both training groups and the decrease in LDL levels in the SO group may be the result of training intensity as previous studies have indicated that high intensity work load impacts lipoprotein levels much more than low intensity work load [27,28].

The results of our study are in agreement with others who reported favorable alterations in apoA-I and LDL levels following specified periods of exercise training [16,20,22]. Body fat percentages were measured throughout the study to account for such changes since body fat percentage is a strong predictor of CVD in children [29]. Changes in BMI and body fat percentage often result in alterations of the lipoprotein profiles [30]. Previous studies in adults have shown favorable alterations in blood lipid and lipoprotein profiles following a controlled exercise intervention [31,32]. For example, Holme *et al.*[33] suggested that exercise training may directly reduce the atherogenicity of lipoproteins via decreasing both apoB and the apoB/apoA-I ratio. Kelley *et al.*[34] concluded that aerobic exercise decreases the levels of both TC and TG, and increases the level of HDL in males 18 years and older. However, few conclusive exercise training reports on adolescent males have been published. The current results are consistent with Ben Ounis *et al.*[16], who demonstrated a significant increase in apoA-I levels for 12–14 year old children running 90 min/day, four days per week, for an 8-week period (Figure 2). Moreover, increases in both apoA-I and HDL have been associated with exercise and subsequent reductions in CVD risk factors. The benefit of HDL is that there is a greater amount of un-esterified cholesterol from the periphery and greater excretion of cholesterol via the liver. The un-esterified cholesterol is removed from cell membranes by apoA-I. Therefore, an increase in the amount of HDL and apoA-I will result in an increased clearance of cholesterol from the periphery and bloodstream [35,36]. Raitakari *et al*. illustrated that with high amounts of physical activity, LDL and apoB levels decrease while HDL levels increase [37]. Since increased levels of HDL, apoA-I, apoA-I/apoB ratio as well as a decreased LDL level were observed in our training subjects, these data suggest that proper swimming and soccer training protocols may potentially reduce the CVD and CHD risk in these adolescent males [16]. It should be noted, however, that neither exercise training program produced significant changes in TC, TG, VLDL and TC/HDL ratio, which is contrary to previous studies [20,22].

Since there is not much literature discussing the effects of exercise in adolescents on apolipoproteins, this study mainly focuses on exercise and its effects on apolipoproteins. However, our research has certain limitations. First, the participants were not on a uniform diet throughout the 12-week period and daily energy intake could not be monitored, which may have affected the results. Although recommendations were made to the participants and their parents in regards to their diet prior to testing, there is no guarantee that these parameters were strictly followed. Secondly, there was no effective control over the amount of exercise for the children outside of the training sessions. Some children may have developed a higher aerobic fitness during the study due to additional exercise outside of the required parameters. Future studies could address baseline fitness values through VO_2_ max testing, etc. Finally, the study was limited to 12 weeks, which may not have been long enough for changes in TC, TG, VLDL, and TC/HDL ratio. Accordingly, it may be that one hour of exercise three days a week is insufficient to produce a significant long term benefit in the complete lipid profile. Nevertheless, our results are encouraging that even 3 months of popular exercise such as swimming and soccer can have some positive effects on certain aspects of the lipid profile.

## Conclusions

The results of the current study illustrate that an increase in plasma apoA-I levels and a decrease in apoB levels can be achieved in 12-week period through performing specific exercise training programs such as swimming and soccer. The favorable changes in LDL, HDL, apoA-I, and apoB levels observed in these training groups demonstrates that regular swimming and soccer exercise training can potentially reduce risk for cardiovascular disease in adolescent males, however possible sustained effects of our interventions are unknown at this point. It should however be noted that the 12-week SW and SO programs were insufficient to lead to favorable changes in TC, TG, and VLDL as well as TC/HDL ratio. Therefore, it may be possible that the SW and SO training programs do not have as great a beneficial effect on adolescent males as compared to adult men.

## Abbreviations

apoA-I: Apolipoprotein A-I; apoB: Apolipoprotein B; BMI: Body mass index; CVD: Cardiovascular disease; C: Control group; CHD: Coronary heart disease; HDL: High-density lipoprotein; HDL-C: High-density lipoprotein-cholesterol; LDL: Low-density lipoprotein; LDL-C: Low-density lipoprotein-cholesterol; RHR: Resting heart rate; SO: Soccer players; SW: Swimmers; TC: Total cholesterol; TG: Triglyceride; VLDL: Very low density lipoprotein.

## Competing interests

The authors declare that they have no competing interests.

## Authors’ contributions

MSK designed the study, performed experiments, and collected the data for analysis. FN supervised MSK during experimentation. RBK, TMB and YR contributed by reviewing and editing the manuscript. WJR contributed by reviewing and editing the manuscript and performed data analysis. LZ contributed by writing and editing the manuscript, performing data analysis, and supervising MSK, YR, and WJR throughout the manuscript preparation. All authors read and approved the final manuscript.

## References

[B1] Buchan DS Ollis S Young JD Thomas NE Cooper SM Tong TK Nie J Malina RM Baker JS The effects of time and intensity of exercise on novel and established markers of CVD in adolescent youth Am J Hum Biol 2011 23 517 526 10.1002/ajhb.21166 21465614

[B2] Cresanta JL Burke GL Downey AM Freedman DS Berenson GS Prevention of atherosclerosis in childhood Pediatr Clin North Am 1986 33 835 858 3737258 10.1016/s0031-3955(16)36076-x

[B3] Kostner GM Czinner A Pfeiffer KH Bihari-Varga M Lipoprotein (a) concentrations as risk indicators for atherosclerosis Arch Dis Child 1991 66 1054 1056 10.1136/adc.66.9.1054 1929512 PMC1793056

[B4] Genest JJ Jr Martin-Munley SS McNamara JR Ordovas JM Jenner J Myers RH Silberman SR Wilson PW Salem DN Schaefer EJ Familial lipoprotein disorders in patients with premature coronary artery disease Circulation 1992 85 2025 2033 10.1161/01.CIR.85.6.2025 1534286

[B5] Lauer RM Connor WE Leaverton PE Reiter MA Clarke WR Coronary heart disease risk factors in school children: the Muscatine study J Pediatr 1975 86 697 706 10.1016/S0022-3476(75)80353-2 1133650

[B6] Hartiala O Magnussen CG Kajander S Knuuti J Ukkonen H Saraste A Rinta-Kiikka I Kainulainen S Kahonen M Hutri-Kahonen N Laitinen T Lehtimäki T Viikari JS Hartiala J Juonala M Raitakari OT Adolescence risk factors are predictive of coronary artery calcification at middle age: the cardiovascular risk in young Finns study J Am Coll Cardiol 2012 60 1364 1370 10.1016/j.jacc.2012.05.045 22981553

[B7] Hager RL Tucker LA Seljaas GT Aerobic fitness, blood lipids, and body fat in children Am J Public Health 1995 85 1702 1706 10.2105/AJPH.85.12.1702 7503350 PMC1615737

[B8] Sothern MS Loftin M Suskind RM Udall JN Blecker U The health benefits of physical activity in children and adolescents: implications for chronic disease prevention Eur J Pediatr 1999 158 271 274 10.1007/s004310051070 10206121

[B9] Freedman DS Cresanta JL Srinivasan SR Webber LS Berenson GS Longitudinal serum lipoprotein changes in white males during adolescence: the Bogalusa heart study Metabolism 1985 34 396 403 10.1016/0026-0495(85)90231-8 3872400

[B10] Orchard TJ Donahue RP Kuller LH Hodge PN Drash AL Cholesterol screening in childhood - does it predict adult hypercholesterolemia - the Beaver county experience J Pediatr 1983 103 687 691 10.1016/S0022-3476(83)80458-2 6631595

[B11] Pekkanen J Linn S Heiss G Suchindran CM Leon A Rifkind BM Tyroler HA Ten-year mortality from cardiovascular disease in relation to cholesterol level among men with and without preexisting cardiovascular disease N Engl J Med 1990 322 1700 1707 10.1056/NEJM199006143222403 2342536

[B12] Castelli WP Abbott RD Mcnamara PM Summary estimates of cholesterol used to predict coronary heart-disease Circulation 1983 67 730 734 10.1161/01.CIR.67.4.730 6825228

[B13] Stergioulas AT Filippou DK Effects of physical conditioning on lipids and arachidonic acid metabolites in untrained boys: a longitudinal study Appl Physiol Nutr Metab 2006 31 432 441 10.1139/h06-020 16900233

[B14] Asztalos BF Roheim PS Milani RL Lefevre M McNamara JR Horvath KV Schaefer EJ Distribution of ApoA-I-containing HDL subpopulations in patients with coronary heart disease Arterioscler Thromb Vasc Biol 2000 20 2670 2676 10.1161/01.ATV.20.12.2670 11116070

[B15] Sarwar N Danesh J Eiriksdottir G Sigurdsson G Wareham N Bingham S Boekholdt SM Khaw KT Gudnason V Triglycerides and the risk of coronary heart disease: 10,158 incident cases among 262,525 participants in 29 Western prospective studies Circulation 2007 115 450 458 10.1161/CIRCULATIONAHA.106.637793 17190864

[B16] Ben Ounis O Elloumi M Makni E Zouhal H Amri M Tabka Z Lac G Exercise improves the ApoB/ApoA-I ratio, a marker of the metabolic syndrome in obese children Acta Paediatr 2010 99 1679 1685 10.1111/j.1651-2227.2010.01920.x 20594189

[B17] Tolfrey K Jones AM Campbell IG The effect of aerobic exercise training on the lipid-lipoprotein profile of children and adolescents Sports Med 2000 29 99 112 10.2165/00007256-200029020-00003 10701713

[B18] Rowland TW Boyajian A Aerobic response to endurance exercise training in children Pediatrics 1995 96 654 658 7567326

[B19] Kelley GA Kelley KS Aerobic exercise and lipids and lipoproteins in children and adolescents: a meta-analysis of randomized controlled trials Atherosclerosis 2007 191 447 453 10.1016/j.atherosclerosis.2006.04.019 16806228 PMC2447165

[B20] Blessing DL Keith RE Williford HN Blessing ME Barksdale JA Blood lipid and physiological responses to endurance training in adolescents Pediatr Exerc Sci 1995 7 192 202

[B21] Szamozi T Hacsek G Szamozi A Popovits I Venekei I Javor A Different cholesterol fractions, LCAT activity and lipid peroxides in the serum of children whose parents had early coronary heart disease Clin Biochem 1988 21 97 99 10.1016/S0009-9120(88)80095-X 3390903

[B22] Tolfrey K Campbell IG Batterham AM Exercise training induced alterations in prepubertal children’s lipid-lipoprotein profile Med Sci Sports Exerc 1998 30 1684 1692 10.1097/00005768-199812000-00005 9861600

[B23] Riepponen P Marniemi J Rautaoja T Immunotubidimetric determination of apolipoproteins A-1 and B in serum Scand J Clin Lab Invest 1987 47 739 747 10.3109/00365518709168939 3685874

[B24] Warnick GR Benderson J Albers JJ Dextran sulfate-Mg2+ precipitation procedure for quantitation of high-density-lipoprotein cholesterol Clin Chem 1982 28 1379 1388 7074948

[B25] Friedewald WT Levy RI Fredrickson DS Estimation of the concentration of low-density lipoprotein cholesterol in plasma, without use of the preparative ultracentrifuge Clin Chem 1972 18 499 502 4337382

[B26] Liu J Sempos C Donahue RP Dorn J Trevisan M Grundy SM Joint distribution of non-HDL and LDL cholesterol and coronary heart disease risk prediction among individuals with and without diabetes Diabetes Care 2005 28 1916 1921 10.2337/diacare.28.8.1916 16043732

[B27] Kraus WE Houmard JA Duscha BD Knetzger KJ Wharton MB McCartney JS Bales CW Henes S Samsa GP Otvos JD Kulkarni KR Slentz CA Effects of the amount and intensity of exercise on plasma lipoproteins N Engl J Med 2002 347 1483 1492 10.1056/NEJMoa020194 12421890

[B28] Paoli A Pacelli QF Moro T Marcolin G Neri M Battaglia G Sergi G Bolzetta F Bianco A Effects of high-intensity circuit training, low-intensity circuit training and endurance training on blood pressure and lipoproteins in middle-aged overweight men Lipids Health Dis 2013 12 131 10.1186/1476-511X-12-131 24004639 PMC3846819

[B29] Williams DP Going SB Lohman TG Harsha DW Srinivasan SR Webber LS Berenson GS Body fatness and risk for elevated blood pressure, total cholesterol, and serum lipoprotein ratios in children and adolescents Am J Public Health 1992 82 358 363 10.2105/AJPH.82.3.358 1536350 PMC1694353

[B30] Tran ZV Weltman A Differential effects of exercise on serum lipid and lipoprotein levels seen with changes in body weight. A meta-analysis JAMA 1985 254 919 924 10.1001/jama.1985.03360070057023 4021025

[B31] Cornelissen VA Fagard RH Effects of endurance training on blood pressure, blood pressure-regulating mechanisms, and cardiovascular risk factors Hypertension 2005 46 667 675 10.1161/01.HYP.0000184225.05629.51 16157788

[B32] Durstine JL Grandjean PW Cox CA Thompson PD Lipids, lipoproteins, and exercise J Cardiopulm Rehabil 2002 22 385 398 10.1097/00008483-200211000-00002 12464825

[B33] Holme I Hostmark AT Anderssen SA ApoB but not LDL-cholesterol is reduced by exercise training in overweight healthy men. Results from the 1-year randomized Oslo diet and exercise study J Intern Med 2007 262 235 243 10.1111/j.1365-2796.2007.01806.x 17645591

[B34] Kelley GA Kelley KS Aerobic exercise and lipids and lipoproteins in men: a meta-analysis of randomized controlled trials J Mens Health Gend 2006 3 61 70 10.1016/j.jmhg.2005.09.003 18645633 PMC2475654

[B35] Miller NE HDL metabolism and its role in lipid transport Eur Heart J 1990 11 Suppl H 1 3 10.1093/eurheartj/11.suppl_H.1 2073908

[B36] Couillard C Despres JP Lamarche B Bergeron J Gagnon J Leon AS Rao DC Skinner JS Wilmore JH Bouchard C Effects of endurance exercise training on plasma HDL cholesterol levels depend on levels of triglycerides: evidence from men of the health, risk factors, exercise training and genetics (HERITAGE) family study Arterioscler Thromb Vasc Biol 2001 21 1226 1232 10.1161/hq0701.092137 11451756

[B37] Raitakari OT Taimela S Porkka KV Telama R Valimaki I Akerblom HK Viikari JS Associations between physical activity and risk factors for coronary heart disease: the cardiovascular risk in young Finns study Med Sci Sports Exerc 1997 29 1055 1061 10.1097/00005768-199708000-00011 9268963

